# Effective cardiac resynchronization therapy for an adolescent patient with dilated cardiomyopathy seven years after mitral valve replacement and septal anterior ventricular exclusion

**DOI:** 10.1186/1749-8090-5-47

**Published:** 2010-06-03

**Authors:** Takahiro Mima, Shiro Baba, Noritaka Yokoo, Shinji Kaichi, Takahiro Doi, Hiraku Doi, Toshio Heike

**Affiliations:** 1 Department of Pediatrics, Graduate School of Medicine, Kyoto University, Kyot Japan; 2 Department of Cardiology, Graduate School of Medicine, Kyoto University, Kyot Japan

## Abstract

Cardiac resynchronization therapy (CRT) is a new treatment for refractory heart failure. However, most heart failure patients treated with CRT are middle-aged or old patients with idiopathic or ischemic dilated cardiomyopathy. We treated a 17 year 11 month old girl with dilated cardiomyopathy after mitral valve replacement (MVR) and septal anterior ventricular exclusion (SAVE). Seven years after the SAVE procedure, she presented complaining of palpitations and general fatigue with normal activity. Her echocardiogram showed reduced left ventricular function. Despite of optimal medical therapy, her left ventricular function continued to decline and she experienced regular arrhythmias such as premature ventricular contractions. We thus elected to perform cardiac resynchronization therapy with defibrillator (CRT-D). After CRT-D, her clinical symptoms improved dramatically and left ventricular ejection fraction (LVEF) improved from 31.2% to 51.3% as assessed by echocardiogram. Serum BNP levels decreased from 448.2 to 213.6 pg/ml. On ECG, arrhythmias were remarkably reduced and QRS duration was shortened from 174 to 152 msec. In conclusion, CRT-D is an effective therapeutic option for adolescent patients with refractory heart failure after left ventricular volume reduction surgery.

## Background

Left ventricular volume reduction therapy has been an effective treatment modality for end-staged dilated cardiomyopathy. Some patients, however, suddenly worsen due to leading to heart failure. To address asynchrony, cardiac resynchronization therapy (CRT) was developed and is recommended by the AHA 2009 guidelines [1]. More than 4000 heart failure patients with ventricular asynchrony have been evaluated in randomized controlled trials of optimal medical therapy alone versus optimal medical therapy plus CRT. CRT has resulted in significant improvements in the quality of life, functional class, exercise tolerance, and ejection fraction [2-5] in patients with refractory heart failure. But most patients are middle-aged and older adults with ischemic heart disease. Moreover, there are no guidelines for pediatric patients. Here we report the successful use of CRT in an adolescent girl after septal anterior ventricular exclusion (SAVE) procedure during childhood.

## Case Presentation

In December 2009, a 17 year, 11 month old adolescent girl with history of worsening dilated cardiomyopathy after mitral valve replacement (MVR) and septal anterior ventricular exclusion (SAVE) was admitted to our hospital for the evaluation for cardiac resynchronization therapy (CRT).

At two months of age, a heart murmur was noted on examination and one year later, she was diagnosed with congenital mitral valve stenosis (MS) and mitral valve regurgitation (MR). Despite optimal medical therapy (digitoxin and diuretics), her left ventricular end-diastolic diameter (LVDd) gradually increased and her MR worsened. She underwent MVR at age six, but the cardiac function deteriorated and LVDd progressively increased. At age 11 years and 1 month, she went into a cardiogenic shock and emergently underwent SAVE and a second MVR procedure emergently. She successfully recovered from cardiogenic shock and the physical activity improved from New York Heart Association (NYHA) class IV to class II [6].

Her cardiac function has remained stable for six years following the SAVE procedure. In June 2008, six years after the SAVE and second MVR procedures, she developed palpitations and general fatigue with regular activity. The LVDd was again dilated, and the left ventricular ejection fraction (LVEF) decreased. In the course of six months prior to her hospitalization, the serum BNP increased from 148.6 pg/ml to 493.1 pg/ml. In addition, QRS duration suddenly widened from 126 msec to 182 msec. Tissue doppler echocardiogram confirmed a clear asynchrony. We recommended CRT placement given her worsening heart failure following left ventricular volume reduction therapy.

**Figure 1:**
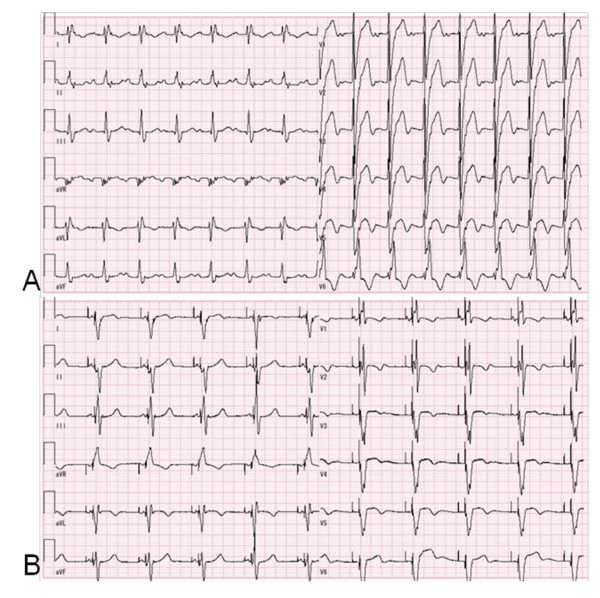
**ECG before and after the CRT placement**. Before the CRT placement (A), QRS duration was prolonged as 174 msec. In addition, ECG showed the prolonged PR interval and complete left bundle branch block (CLBBB). After the CRT placement (B), the QRS duration was shortened to 152 msec. In addition, PR interval was shortened and CLBBB changed to complete right bundle branch block (CRBBB).

At the time of admission, her body weight and height was 33.0 kg and 143.0 cm. The chest X-ray revealed significant increases in cardio-thoracic ratio (CTR) (70.6%) and pulmonary congestion. Echocardiogram showed LVDd of 59.3 mm (134.2% of normal), and reduced LVEF. LVEF was also measured by a cardiac MRI, and was estimated to be 23.7%. Serum BNP was again elevated at 448.4 pg/ml. Her ECG showed widened QRS duration of 174 msec and complete left bundle branch block (CLBBB) (Figure 1A). We concluded that cardiac asynchrony was probably responsible for her worsening heart failure. She was immediately scheduled for CRT placement.

**Figure 2:**
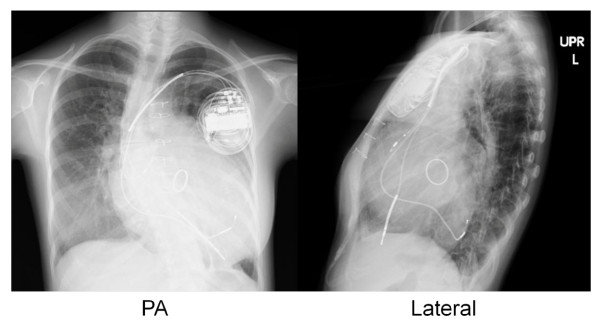
**The chest X-ray (PA and Lateral projection) after the CRT placement**.

**Figure 3:**
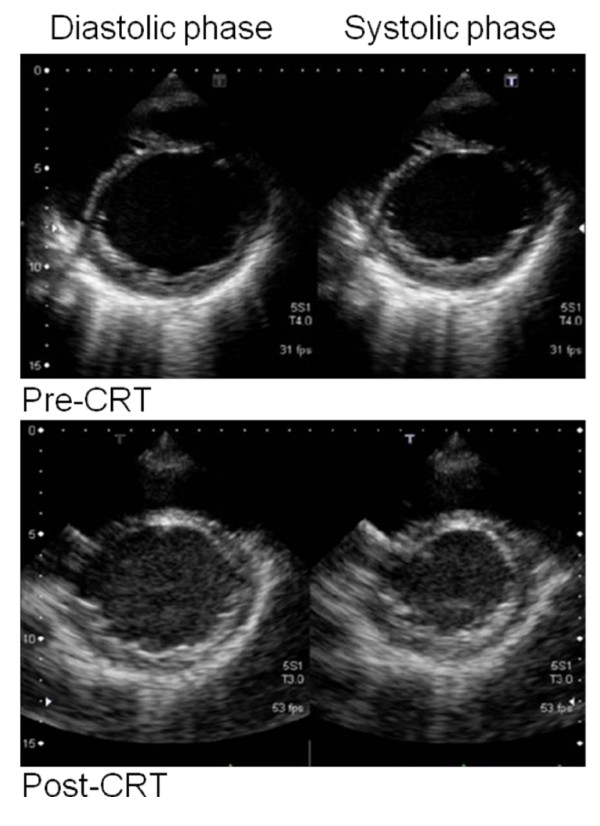
**Echocardiogram before and after the CRT placement**. Each panel shows the short axis view of the LV cavity. Upper panels: The LVDd was 59.3 mm and LVEF was 31.2% before the CRT placement. Lower panels: The LVDd was 61.3 mm and LVEF was 51.3% after the CRT placement.

On the third day of hospitalization, CRT with defibrillator (CRT-D) implantation was performed by transvenous approach. We chose CRT-D, Medtronic Concerto C154DWK, because she experienced arrhythmias such as premature ventricular contractions (PVC), non-sustained ventricular tachycardia and atrial flutter. Before the CRT implantation, we assessed asynchrony by tissue doppler echocardiogram and determined the most delayed site of the whole wall movement. We targeted the most delayed site as the optimal pacing site. But the strong degeneration of cardiac muscles restricted the possible pacing site. We placed an atrial lead at the right appendage and a RV lead at the apex. A LV lead was placed at the lateral wall of the coronary sinus because there was the possibility that the main trunk of the coronary sinus was occluded during the SAVE procedure assessed by contrast medium. We show the final pacing site by the chest X-ray (Figure 2). The pacemaker mode was DDD 60-130 bpm biventricular pacing. While testing the implantable cardioverter-defibrillator (ICD), a 10 J defibrillation was administered for ventricular fibrillation. All segmental max delay and all segmental standard deviation improved from 140 msec to 86 msec and 44 msec to 26 msec, respectively, by tissue doppler echocardiogram. Three months after the CRT-D implantation, the LVEF improved from 31.2% to 51.3% (Figure 3) and the serum BNP levels decreased from 448.2 to 213.6 pg/ml. The QRS duration was shortened from 174 to 152 msec (Figure 1B), and arrhythmias were extremely reduced. By a Holter monitoring, the number of PVCs reduced from 3625 to 127 and double-barreled PVCs reduced from 101 to 1 per 24 hours. The physical activity improved remarkably and the NYHA classification improved from class III to class II for around one year. She was able to resume her previous level of activity.

## Conclusion

CRT has become an accepted method for treating refractory heart failure in patients with idiopathic or ischemic dilated cardiomyopathy associated with electromechanical asynchrony. In the current AHA guidelines, CRT is a class I (level of evidence A) therapy for patients with a LVEF less than or equal to 35% and a QRS duration greater than or equal to 120 msec who are symptomatic (NYHA functional Class III or IV) despite optimal recommended medical therapy [7]. At the time of our patient's admission, she meets all these criteria despite optimal medical therapy. We thus thought that she was an appropriate candidate for CRT.

CRT may be performed with or without defibrillator. In the CARE-HF (Cardiac Resynchronization in Heart Failure) trial, a randomized controlled trial comparing optimal medical therapy alone with optimal medical therapy plus CRT without a defibrillator, CRT significantly reduced the combined risk of death of any cause or unplanned hospital admission for major cardiovascular events (analyzed as time to first event) by 37% [8]. In the COMPANION (Comparison of Medical Therapy, Pacing, and Defibrillation in Heart Failure) trial, directly compared pacing with CRT-D and CRT without defibrillation with optimal medical therapy, only CRT-D reduced sudden cardiac death (SCD) [9,10]. Although there was insufficient evidence to conclude that CRT alone was inferior to CRT-D, we selected CRT-D in our patient because of her repeated arrhythmias.

The efficacy of CRT in the young and in those with congenital heart disease (CHD) has not yet been established because the vast majority of patients included in randomized clinical studies of CRT have cardiomyopathy of ischemic or idiopathic etiology and most patients are middle-aged and older adults. Although there are no prospective trial data, retrospective series show that CRT is similarly effective for managing asynchrony-associated heart failure in the younger population as it is for treating adults with ischemic and idiopathic dilated cardiomyopathy [11,12]. And our case demonstrates that CRT is a useful adjunct in the treatment of heart failure in the young after the LV volume reduction surgery. In addition, CRT has been discussed as an alternative to cardiac transplantation in advanced heart failure. Bert Hansky *et al*. demonstrate that CRT is a reliable therapeutic option for the long-term treatment of end-stage heart failure and LV asynchrony [13]. In many countries, cardiac transplantation is difficult because donors are particularly rare. This is one of the reasons why we elected to perform CRT. CRT also may become a bridge to transplant that offers extended patient longevity and improved quality of life to younger patients with CHD and end-stage heart failure [14]. Our case demonstrates the merit of such a concept.

We conclude that CRT is a useful adjunct in the treatment of heart failure in adolescents after the SAVE procedure. We propose that CRT is a promising modality for the treatment of refractory heart failure with asynchronous LV wall motion. Further studies are needed to determine the indication, effectiveness, and the long-term benefits of this therapy in the pediatric population.

## Consent

Written informed consent was obtained from this patient and her mother for publication of this case report and any accompanying images. A copy of the written consent is available for review by the Editor-in-Chief of this journal.

## Abbreviations

CRT: Cardiac Resynchronization Therapy; SAVE: Septal Anterior Ventricular Exclusion; CRT-D: Cardiac Resynchronization Therapy with Defibrillator; MVR: Mitral Valve Replacement; MS: Mitral valve Stenosis; MR: Mitral valve Regurgitation; LVDd: Left Ventricular End-Diastolic Diameter; NYHA: New York Heart Association; LVEF: Left Ventricular Ejection Fraction; CTR: Cardio-Thoracic Ratio; CLBBB: Complete Left Bundle Branch Block; PVC: Premature Ventricular Contractions; ICD: Implantable Cardioverter-Defibrillator; CARE-HF: Cardiac Resynchronization in Heart Failure; COMPANION: Comparison of Medical Therapy, Pacing, and Defibrillation in Heart Failure; SCD: Sudden Cardiac Death; CHD: Congenital Heart Disease; CRBBB: Complete Right Bundle Branch Block

## Competing interests

The authors declare that they have no competing interests.

## Authors' contributions

TM was an attending physician in the pediatric ward in Kyoto university hospital, and wrote most part of this manuscript. SB is an attending physician in the pediatric outpatient clinic in Kyoto university hospital, and gave most comments for this manuscript. NY is an attending physician in the pediatric ward in Kyoto university hospital. SK is an attending physician in the pediatric outpatient clinic in Kyoto university hospital. TD is an attending physician in the cardiovascular outpatient clinic in Kyoto university hospital. HD is an attending physician in the pediatric outpatient clinic in Kyoto university hospital. TH is a general supervisor of this manuscript.

## Authors' information

TM is a graduate student and a pediatric cardiologist in charge in a pediatric ward of Kyoto university hospital. SB is an assistant professor and a pediatric cardiologist in charge in a pediatric ward of Kyoto university hospital. NY is a graduate student and a pediatric cardiologist in charge in a pediatric ward of Kyoto university hospital. SK is an assistant professor and a pediatric cardiologist in a pediatric ward of Kyoto university hospital. TD is an assistant professor and a cardiologist in charge in a cardiovascular ward of Kyoto university hospital. HD is an assistant professor and a pediatric cardiologist in charge in a pediatric ward and an outpatient clinic of Kyoto university hospital. TH is a professor of the pediatrics department in Kyoto university hospital. He is a supervisor of this manuscript.

## References

[B1] Hunt SA Abraham WT Chin MH Feldman AM Francis GS Ganiats TG Jessup M Konstam MA Mancini DM Michl K Oates JA Rahko PS Silver MA Stevenson LW Yancy CW 2009 Focused Update Incorporated Into the ACC/AHA 2005 Guidelines for the Diagnosis and Management of Heart Failure in Adults A Report of the American College of Cardiology Foundation/American Heart Association Task Force on Practice Guidelines: developed in collaboration with the International Society for Heart and Lung Transplantation Circulation 2009 119 14 e391 479 10.1161/CIRCULATIONAHA.109.192065 19324966

[B2] Higgins SL Hummel JD Niazi IK Giudici MC Worley SJ Saxon LA Boehmer JP Higginbotham MB Marco TD Foster E Yong PG Cardiac resynchronization therapy for the treatment of heart failure in patients with intraventricular conduction delay and malignant ventricular tachyarrhythmias J Am Coll Cardiol 2003 42 1454 1459 10.1016/S0735-1097(03)01042-8 14563591

[B3] Abraham WT Fisher WG Smith AL Delurgio DB Leon AR Loh E Kocovic DZ Packer M Clavell AL Hayes DL Ellestad M Messenger J Cardiac resynchronization in chronic heart failure N Engl J Med 2002 346 1845 1853 10.1056/NEJMoa013168 12063368

[B4] Young JB Abraham WT Smith AL Leon AR Lieberman R Wilkoff B Canby RC Schroeder JS Liem LB Hall S Wheelan K Combined cardiac resynchronization and implantable cardioversion defibrillation in advanced chronic heart failure: the MIRACLE ICD Trial JAMA 2003 289 2685 2694 10.1001/jama.289.20.2685 12771115

[B5] McAlister FA Stewart S Ferrua S McMurray JJJV Multidisciplinary strategies for the management of heart failure patients at high risk for admission: a systematic review of randomized trials J Am Coll Cardiol 2004 44 810 819 15312864 10.1016/j.jacc.2004.05.055

[B6] Baba S Doi H Ikeda T Komeda M Nakahata T A long-term follow-up of a girl with dilated cardiomyopathy after mitral valve replacement and septal anterior ventricular exclusion J Cardiothorac Surg 2009 4 53 55 10.1186/1749-8090-4-53 19775464 PMC2758862

[B7] Epstein AE DiMarco JP Ellenbogen KA Estes NA Freedman RA Gettes LS Gillinov AM Gregoratos G Hammill SC Hayes DL Hlatky MA Newby LK Page RL Schoenfeld MH Silka MJ Stevenson LW Sweeney MO Smith SC Jr Jacobs AK Adams CD Anderson JL Buller CE Creager MA Ettinger SM Faxon DP Halperin JL Hiratzka LF Hunt SA Krumholz HM Kushner FG Lytle BW Nishimura RA Ornato JP Page RL Riegel B Tarkington LG Yancy CW ACC/AHA/HRS 2008 Guidelines for Device-Based Therapy of Cardiac Rhythm Abnormalities: a report of the American College of Cardiology/American Heart Association Task Force on Practice Guidelines (Writing Committee to Revise the ACC/AHA/NASPE 2002 Guideline Update for Implantation of Cardiac Pacemakers and Antiarrhythmia Devices): developed in collaboration with the American Association for Thoracic Surgery and Society of Thoracic Surgeons Circulation 2008 117 21 e350 408 10.1161/CIRCUALTIONAHA.108.189742 18483207

[B8] Cleland J Daubert J Erdman E Freemantle N Gras D Kappenberger L Tavazzi L for the Cardiac Resynchronization-Heart Failure (CARE-HF) Study Investigators The effect of cardiac resynchronization on morbidity and mortality in heart failure N Engl J Med 2005 352 1539 1549 10.1056/NEJMoa050496 15753115

[B9] Bristow MR Saxon LA Boehmer J Krueger S Kass DA Marco TD Carson P DiCarlo L DeMets D White BG DeVries DW Feldman AW for the Comparison of Medical Therapy, Pacing, and Defibrillation in Heart Failure (COMPANION) Investigators Cardiac-resynchronization therapy with or without an implantable defibrillator in advanced chronic heart failure N Engl JMed 2004 350 2140 2150 10.1056/NEJMoa032423 15152059

[B10] Carson P Anand I O'Connor C Jaski B Steinberg J Lwin A Lindenfeld J Ghali J Barnett JH Feldman AM Bristow MR Mode of death in advanced heart failure: the Comparison of Medical, Pacing, and Defibrillation Therapies in Heart Failure (COMPANION) trial J Am Coll Cardiol 2005 46 2329 2334 10.1016/j.jacc.2005.09.016 16360067

[B11] Dubin AM Janoušek J Rhee E Strieper MJ Cecchin F Law IH Shannon KM Resynchronization therapy in pediatric and congenital heart disease patients: An international multicenter study J Am Coll Cardiol 2005 46 2277 2283 10.1016/j.jacc.2005.05.096 16360058

[B12] Janousek J Gebauer RA Cardiac Resynchronization Therapy in Pediatric and Congenital Heart Disease PACE 2008 31 Suppl 1 21 23 10.1111/j.1540-8159.2008.00949.x 18226029

[B13] Hansky B Vogt J Zittermann A Güldner H Heintze J Schulz U Horstkotte D Tenderich G Körfer R Cardiac Resynchronization Therapy: Long-Term Alternative to Cardiac Transplantation? Ann Thorac Surg 2009 87 432 439 10.1016/j.athoracsur.2008.09.071 19161754

[B14] Moak JP Hasbani K Ramwell C Freedenberg V Berger JT DiRusso G Callahan P Dilated cardiomyopathy following right ventricular pacing for AV block in young patients: resolution after upgrading to biventricular pacing systems J Cardiovasc Electrophysiol 2006 17 1068 1071 10.1111/j.1540-8167.2006.00565.x 16989648

